# Stabilised Hyaluronic Acid (sHA) gel as a novel marker for breast cancer tumour bed cavity: Surgical feasibility

**DOI:** 10.1016/j.ctro.2024.100745

**Published:** 2024-02-19

**Authors:** Janice Yeh, Grace Chew, Suat Li Ng, Wei Ming Ooi, Su-Wen Loh, Anthony Hyett, Tristan Leech, Elaine Bevington, Jenny Huynh, Jenny Sim, Farshad Foroudi, Sweet Ping Ng, Michael Chao

**Affiliations:** a Department of Radiation Oncology, Olivia Newton-John Cancer Wellness & Research Centre, Austin Health, Victoria, Australia; b Department of Radiation Oncology, Peter MacCallum Cancer Centre, Victoria, Australia; c Department of Medical Imaging and Radiation Sciences, Monash University, Clayton, Victoria, Australia; d Department of Breast Surgery, Austin Health, Victoria, Australia; e University of Melbourne, Victoria, Australia; f Genesis Care, Ringwood Private Hospital, Victoria, Australia

**Keywords:** Breast cancer tumor bed radiotherapy, Hyaluronic acid gel fiducial marker, Surgical feasibility

## Abstract

**Introduction:**

Consistent delineation of the breast conserving surgery (BCS) tumour bed (TB) for breast cancer remains a challenge for radiation oncologists. Accurate delineation allows for better local control and reduces toxicity when planning partial breast or TB boost radiation therapy (RT).

**Methods:**

In the operating theatre (OT) breast surgeons inserted stabilised hyaluronic acid (sHA) gel as small drops approximately one cm into the walls surrounding the resection cavity. Surgical feasibility was determined by the rate of successful sHA gel insertion procedure, the ease of insertion as rated by surgeons, the time required for insertion procedure, the quantity used, and any adverse events (AE) relating to sHA gel insertion.

**Results:**

Thirty-five patients were enrolled. All patients underwent sHA gel insertion successfully. The procedure added a median of 2.8 min to the OT time and was rated as ‘easy’ in 89 % of patients. There were no immediate AE in OT. Five (14 %) patients experienced a grade 2 or higher AE. Three of the five patients were prescribed oral antibiotics for breast infection. Two of the five patients experienced a grade 3 AE – haematoma which required evacuation in OT day 1 post-BCS, and infected seroma which required drainage and washout in OT 2 months post-BCS. All five patients recovered and underwent the planned adjuvant therapies for their BC. The AE data reflects common risks with standard BCS and are not clearly attributed to sHA gel insertion alone.

**Conclusion:**

We show that sHA gel is surgically feasible as a marker to help define the TB cavity for post-BCS adjuvant MRI-based RT planning.

## Highlights

• Hyaluronic acid gel can be used to mark breast cancer tumour bed for MRI-based RT planning.
• Hyaluronic acid gel is quick and easy to use.
• Hyaluronic acid gel is safe to use.

## Introduction

Adjuvant radiation therapy (RT) following breast conserving surgery (BCS) is the current standard of care in breast conserving therapy of early breast cancer because it reduces the locoregional recurrence rate and risk of breast cancer death [1]. When planning breast RT, in particular delineation of the tumour bed (TB) for boost RT or partial breast irradiation (PBI), clinical landmarks such as the surgical scar and induration area, or ultrasound evaluation, have been shown to be unreliable surrogates for the TB localisation when compared to titanium surgical clips (SC) or gold fiducial markers placed at the excisional cavity wall intra-operatively [2], [3], [4].

The presence of fiducial markers has been shown to reduce interobserver variability in the delineation of the TB, when compared to CT simulation planning scans without fiducial markers [5], [6]. These markers also serve to provide a reference point for image-guidance during RT treatment delivery [7], [8]. The number and placement of SC to allow for accurate TB localisation required in the UK IMPORT LOW trial [9] as recommended by the trial management group was six titanium clips, positioned in the medial, lateral, superior, inferior, deep (pectoral fascia), and anterior (close to suture line) points of the TB [10]. Similarly, the Canadian Locally Advanced Breast Cancer National Consensus Group has published consensus recommendations which include intraoperative placement of a minimum of four SC in BCS, to assist with TB delineation and adjuvant RT planning [11]. While SC help improve consistency with TB delineation, they also have limitations, such as confusion between those used for TB marking versus for haemostasis [12].

Several novel fiducial markers aiming to replace or supplement SC have been reported in the literature. Barrigel® (Palette Life Sciences, USA) is a sterile, transparent, biodegradable gel of non-animal stabilised hyaluronic acid (sHA) at a concentration of 20 mg/mL in phosphate buffered saline, stored in a glass syringe. It has been registered on the Australian Register of Therapeutic Goods since July 2020 as a RT protection spacer, “used to increase the distance between the prostate and the anterior rectal wall, with the intent to decrease radiation dose delivered to the rectum when treating prostate cancer with radiation” [13]. sHA gel can also be used as a tissue fiducial marker that is known to be visible on MRI, which can be helpful as the role of MRI simulation and MR linear accelerator therapy are increasingly adopted in the radiation oncology community.

The aim of this single arm prospective study is to report the surgical feasibility of sHA gel as a novel marker to help guide delineation of the post-BCS TB for RT planning in a single centre. Further work on TB delineation and interobserver variability comparing sHA gel versus surgical clips is in progress and will be reported in a future paper.

## Materials and methods

### Study population

Female patients with clinically node negative, unifocal early breast cancer, measuring a maximum diameter of 3 cm, planned for wide local excision (WLE) and sentinel lymph node biopsy were eligible for the study. The breast carcinoma could be of any grade, oestrogen receptor (ER), progesterone receptor (PR), and human epidermal growth factor receptor 2 (HER-2) status. Exclusion criteria included patients with allergy to hyaluronic acid, contraindication to magnetic resonance imaging (MRI), or who are unsuitable for RT (eg. pregnancy, previous ipsilateral breast RT). Informed consent was obtained from all patients and/or their legal guardian(s).

### Fiducial marker insertion

**Fig. 1:**
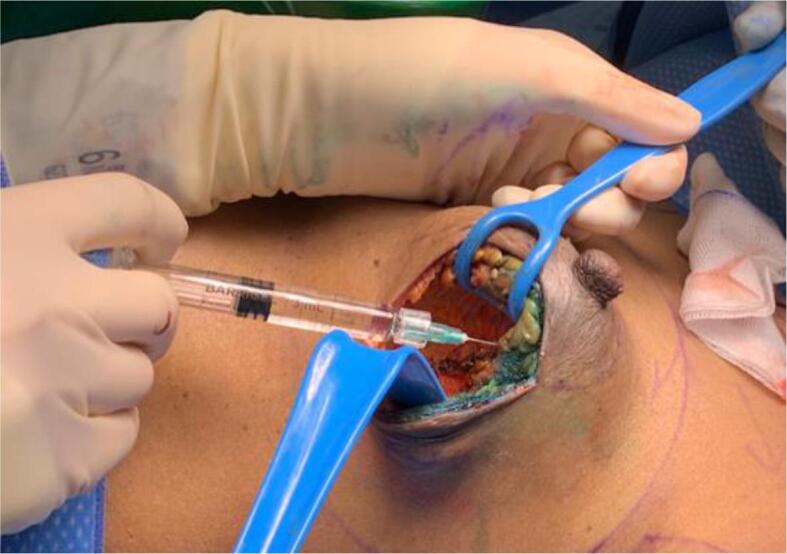
Photograph of intra-operative tumour bed marking with stabilised hyaluronic acid (sHA) gel.

Markers for each patient included the placement of SC after the WLE and insertion of sHA gel. Up to 6 SC were inserted onto the four radial, the deep and the superficial margins of the excision cavity. sHA gel was inserted with the goal to mark the deep and radial margins of excision cavity. This involved drops (approximately 0.3–0.5 ml (mL)) injected using a sterile 21–23 gauge needle, starting at the base, injected just under the pectoral fascia at 4 points of the compass, indicating the deep margin. Further 4 drops were injected approximately 1 cm interval above the base plane, also at 4 points of the compass, but at 1 cm into the cavity wall, into the adjacent breast tissue. This was repeated at further 1 cm intervals above the previous insertion plane until the skin was reached. Fig. 1 shows a photograph of sHA gel insertion protocol in the operating theatre.

As the standard SC marker were also placed, the investigational marker sHA gel was not used to influence the subsequent clinical care of the patients.

### Evaluation of endpoints and statistical analysis

Endpoints of this paper focused on the surgical feasibility of the use of sHA gel as a fiducial marker, including the rate of successful sHA gel insertion procedure (insertion performed per protocol), the surgeons’ rating of the ease of insertion procedure using a three-point Likert scale (easy, moderate or difficult), and time required for the insertion (including opening of additional syringes and local haemostasis procedures as required), and therefore time added to surgery. Adverse events (AE) related to sHA gel insertion from time of insertion to start of adjuvant RT were graded using common terminology criteria for adverse events (CTCAE) version 5.

Descriptive statistics for the study participants are presented as frequencies and percentages of patients in each category for categorical variables, with continuous variables presented either as mean if they are normally distributed, or median if they are non-normally distributed. Surgical feasibility is defined as ≥90 % of patients who undergo sHA gel insertion successfully and were rated by surgeons to have ‘easy’ or ‘moderate’ gel insertion procedures.

All methods were carried out with the approval of the institutional human research ethics committee of Austin Health (HREC/68327/Austin-2020) and Monash University. All methods were carried out in accordance with relevant guidelines and regulations.

## Results

Between June 2021 and August 2022, 35 patients consented to participate in the study. The patients’ baseline characteristics are summarised in Table 1. The average age at diagnosis was 61.3 years old. Majority of patients (84 %) had a tumour less than 2 cm in diameter on pre-operative assessment and all were clinically node negative.

**Table 1:** Demographic and clinicopathologic characteristics of patients.

Characteristic	Variable	Value
Age	Range (years)	42–82 (mean 61.3)
Laterality	Left	19 (54 %)
	Right	16 (46 %)
Quadrant	Upper outer quadrant (UOQ)	20 (57 %)
	Upper inner quadrant (UIQ)	8 (23 %)
	Lower outer quadrant (LOQ)	4 (11 %)
	Lower inner quadrant (LIQ)	3 (9 %)
Clinical Tumour stage (AJCC 8th Ed)	cT1a (1–5 mm)	1 (2.9 %)
	cT1b (>5–10 mm)	10 (28.5 %)
	cT1c (>10–20 mm)	17 (48.6 %)
	cT2 (>20–50 mm)	7 (20.0 %)
Clinical Nodal stage	cN0	35 (100 %)
Histology	Invasive ca NST	27 (77.1 %)
	Invasive lobular ca	4 (11.4 %)
	Other	4 (11.4 %)
Pathological Tumour stage (AJCC 8th Ed)	pT1a (1–5 mm)	1 (3 %)
	pT1b (>5–10 mm)	5 (14 %)
	pT1c (>10–20 mm)	16 (46 %)
	pT2 (>20–50 mm)	13 (37 %)
Pathological Nodal stage	pN0	29 (83 %)
	pN1a	5 (14 %)
	pN2a	1 (3 %)
Grade	1	9 (26 %)
	2	19 (54 %)
	3	7 (20 %)

All 35 patients underwent WLE and SLNB performed by eight specialist breast surgeons at one institution. The breakdown of number of procedures each of the eight surgeons performed is shown in Table 2, together with the numbers of oncoplastic mammoplasty procedures following WLE. Level 2 vol displacement oncoplastic manoeuvres were performed in 34 % of the cases.

**Table 2:** Surgical data.

	Variable	Value
Specialist breast surgeon	Surgeon A	4 (11.4 %)
	Surgeon B	9 (25.7 %)
	Surgeon C	7 (20 %)
	Surgeon D	2 (5.7 %)
	Surgeon E	3 (8.6 %)
	Surgeon F	6 (17.1 %)
	Surgeon G	2 (5.7 %)
	Surgeon H	2 (5.7 %)

Level 2 volume displacement oncoplastic wide local excision (WLE)	Yes	11 (31 %)
	No	24 (69 %)

All enrolled patients underwent sHA gel insertion successfully. The volume of sHA gel used per patient, total time from start to finish of sHA gel insertion, and the surgeons’ ratings of ease of the procedure is summarised in Table 3. A median of 3 mL of sHA gel was used and the insertion was performed fast, with a median time of 2.8 min and a maximum of 9 min. Surgical feasibility as defined in this study was met as 100 % of patients enrolled had sHA gel insertion per protocol instructions and were rated by surgeons to have been ‘easy’ or ‘moderate’ procedures.

**Table 3:** Stabilised hyaluronic acid (sHA) gel insertion data.

	Variable	Value
Total sHA gel used per patient	Range of volume (mL)	1.25–9 mL (median 3 mL)
Total time from first to final drop of sHA gel	Range of time (minutes)	1–9 min (median 2.8 min)
Ease of insertion (surgeons’ rating)	Easy	31 (89 %)
	Moderate	4 (11 %)
	Difficult	0

Two of 35 (6 %) patients had a CTCAE grade 3 AE recorded. One (3 %) of them had a haematoma which underwent evacuation in the operating theatre day 1 after their BCS. She was not on regular anticoagulation medication but did receive a prophylactic dose of enoxaparin for deep vein thrombosis (DVT) prophylaxis, administered at one hour from start of surgery. She has since recovered well and completed planned adjuvant chemotherapy and adjuvant RT for her pT1N0 grade 3 ER/PR negative, HER2 amplified breast cancer without any complications. It is uncertain that this event was related to the sHA gel insertion given that haematomas can occur after breast surgery without sHA gel insertion. The other patient (3 %) had an infected breast and axillary seroma which required drainage and washout in the operating theatre. However, this occurred 2 months following sHA gel insertion, in the setting of subsequent axillary dissection surgery, outpatient drainage of the recurrent seroma, and a neutropenic episode from cycle 1 of chemotherapy. She has since recovered well and completed planned adjuvant chemotherapy and adjuvant RT for her pT2N2aM0 grade 3 ER positive PR negative HER2 amplified breast cancer. Given the timeline and other more likely contributing factors, it was determined that this AE would unlikely be related to the sHA gel insertion.

Three of 35 (9 %) patients had CTCAE grade 2 AE in the form of mild post-operative infections which were treated with oral antibiotics in the outpatient setting.

## Discussion

**Fig. 2:**
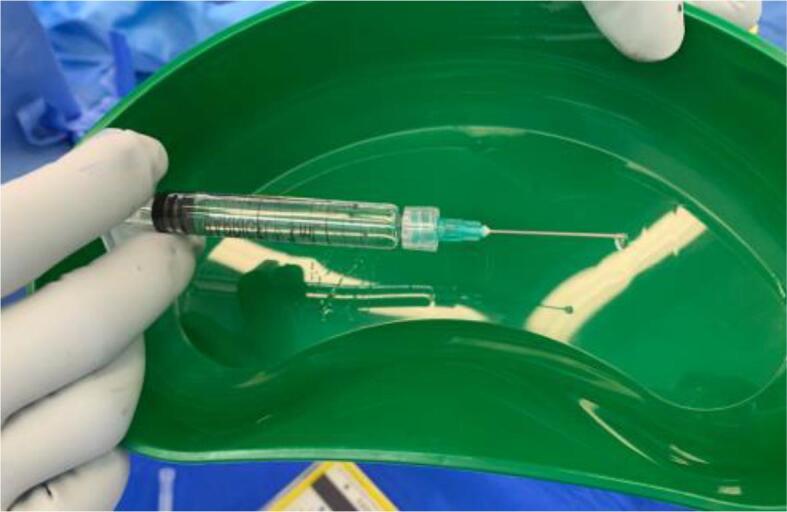
Stabilised hyaluronic acid (sHA) gel syringe.

This is a single arm prospective study investigating the feasibility of inserting sHA gel reporting on surgical data without details on its application in RT planning. We showed that marking the edges of breast cancer TB cavity after WLE using multiple small drops of gel was feasible without difficulty from surgeons’ perspective. The procedure only added a median of under 3 min to overall theatre time and did not interfere with surgeons’ usual techniques including allowing for oncoplastic manoeuvres. The median amount of sHA gel injected was 3 mL, ranging from 1.25 mL to 9 mL. Since the gel is supplied in a sterile 3 mL glass syringe sealed with a plunger and tip cap, the product is ready for immediate use after replacing the tip cap with a 21–23 gauge needle (Fig. 2).

It is uncertain that the AE reported in this study are related to the sHA gel insertion since they are common after breast surgery. A review of 561 patients that underwent WLE at a single institution found that the rate of surgical site complication was 17.9 % in the standard surgery group and 8 % in the oncoplastic surgery group [23]. Another review of 75 patients that underwent oncoplastic breast surgery found an overall clinically relevant postoperative complication rate of 18.7 % [24]. They reported surgical site infection in ten patients (13.3 %) and one patient (1.3 %) in this group of 75 had a haematoma and wound dehiscence. Since the current standard TB marker used in practice is surgical clips, we assume that the AE associated with use of clips would be captured in these data. The total surgical site infection rate in this study was 11 % (n = 4), which despite the small total number of 35 patients, still falls within reported rates in the literature, and thus is comparable to the more common clip markers.

The rates of grade 3 or higher AE in this study is small, with two of thirty-five patients experiencing a CTCAE grade 3 event, noting the overall small total number of patients. The one case of haematoma requiring evacuation in the operating theatre was deemed to be possibly related to the sHA gel insertion, however breast surgery itself is also associated with a low risk of haematoma. This AE completely resolved following the evacuation of haematoma procedure and the patient had no recurrence of bleeding or any other adverse events from the study procedures. The one case of infected seroma requiring drainage and washout in the operating theatre was unlikely related to the sHA gel insertion given the multiple other more likely risk factors as well as the timing.

With multiple randomised controlled trials showing the benefit of adjuvant breast RT following BCS for breast cancer [1] as well as ductal carcinoma in situ (DCIS) [14], in particular PBI [9], [15], [16], [17], [18] and TB boost irradiation [19], accurate and consistent delineation of the TB during RT planning is more important than ever. Potential implications of the RO’s inability to confidently delineate the TB on RT planning scan includes under-coverage of the at-risk area leading to compromised local control rates, over-coverage leading to unnecessary irradiation of normal breast tissue and thus toxicity, or inability to offer PBI to a patient who would have been suitable.

Titanium SC placed at the edges of the resection cavity by breast surgeons is the current standard marker used to provide useful points of reference for ROs to delineate the TB cavity [20]. However, ROs still need to generate an irregular three-dimensional volume based on limited numbers of ‘points’, and this is potentially complicated by the surgeons’ use of SC for non-marking reasons such as haemostasis which can lead to confusion [12]. These issues are exaggerated when oncoplastic manoeuvres are performed when significant tissue flaps and rotations occur. SC can also migrate from initial placement location during surgical closure of surrounding tissue or even post-operatively [21]. Metallic markers can be placed by interventional radiologists following biopsy of ipsilateral breast lesions which are proved to be benign and therefore left in-situ and be challenging to distinguish from TB markers. Despite this, presence of SC to help delineate the TB cavity is still much preferred by all ROs than no markers at all [22]. However, the type of marker can be improved.

The perceived advantage of using sHA gel drops to mark out the TB cavity, is that multiple drops at approximately 1 cm intervals would mean more points of reference for the ROs to delineate the edges appropriate to the specific type of surgery that has occurred. As sHA gel is expected to be bioabsorbable, there would not be concerns of causing future imaging artifact, and patients may not have to accept permanent objects left behind in their breast. The sHA gel would also be a dedicated TB marker, eliminating any confusions that surgical clips can cause when used for non-TB marking reasons. If patients require re-excision of close or positive surgical margins, it can be assumed that some of the sHA gel would also be excised, and additional gel can be inserted at the ‘new’ margin. Using stabilised HA which has an estimated half-life of 6–24 months, it is expected that the markers will stay long enough to remain visible at time of RT, even for patients who may require adjuvant chemotherapy and postponement of adjuvant RT.

**Fig. 3:**
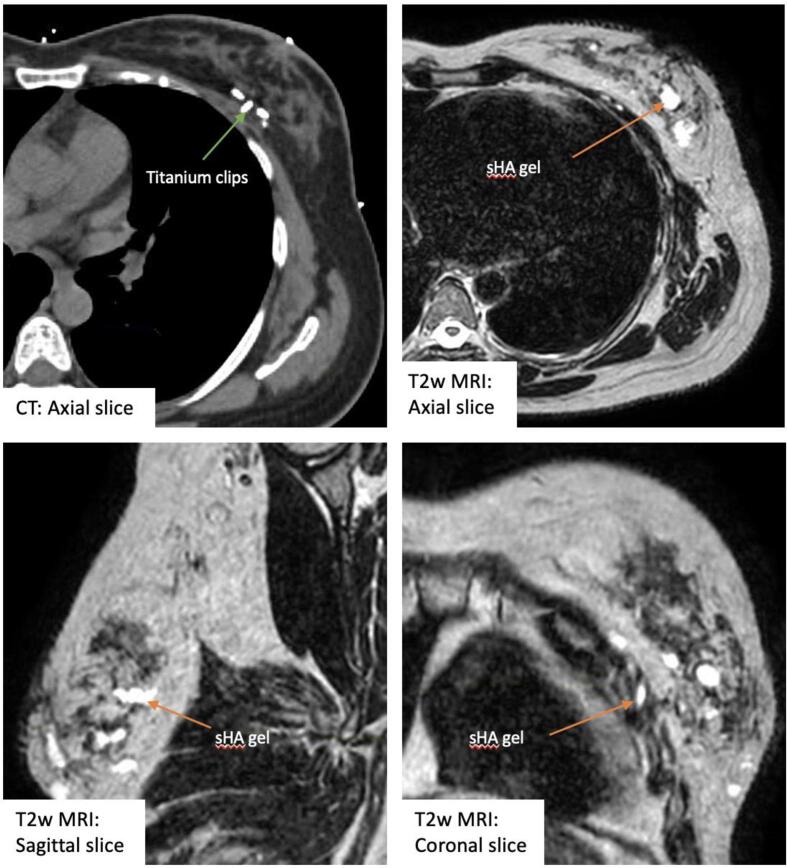
Four-week post-operative computed tomography (CT) and T2-weighted (T2w) magnetic resonance imaging (MRI) scans of a patient with both surgical clips and stabilised hyaluronic acid gel (sHA) gel in-situ.

As sHA gel is not radio-opaque, one limitation is that it can be challenging to visualise and distinguish from normal glandular breast tissue on standard CT planning scans. This would significantly limit its usefulness in CT based RT planning. However, in the era of the use of MRI simulation planning scans, and MRI-guided linear accelerator machines to deliver RT, this characteristic of sHA gel would become more relevant. In our future TB delineation inter-observer variation evaluation study, MRI planning scans are used (Fig. 3). We will be comparing TB delineated using sHA gel and MRI versus clips and CT to establish the gel’s true applicability in clinical practice. Data regarding sHA gel’s expected lifespan and visibility on post-treatment surveillance imaging will also be collected and reported in a future paper.

Several other novel fiducial markers have been investigated to replace or supplement titanium SC to mark the post-WLE TB cavity. Novel markers, including sHA, intended to perform better than SC may likely cost more.

BioZorb® (Focal Therapeutics, Aliso Viejo, CA) is a 3-dimensional device that is implanted at the tumour excision site, consisting of six titanium clips distributed inside a bioabsorbable coil. It is visible on CT simulation planning scans but can still lead to confusion if other SC are used for non-TB marking reasons nearby. BioZorb’s physical structure has been reported to serve as volume replacement during breast cancer surgery which sHA gel cannot. This however leads to the device being palpable during follow-up which can be anxiety inducing for patients and result in additional imaging that would not have been routinely performed [25]. Infection rates associated with BioZorb quoted in the literature were low, 6 % in a retrospective study of 89 patients [25] and 2.1 % in a registry study of 818 patients [26]. Surgical complications (eg. Infection, migration of the device to the axilla, or erosion of the device through nipple-areolar complex [27]sometimes led to device removal with a further surgical procedure for patients. The patients in our study are undergoing longer term follow up for any unexpected future complications, although this is felt less likely given the physical properties and small quantity of sHA gel used (median 3 mL).

Biomarc® Restore (Carbon Medical Technologies, Inc, St. Paul, MN) is a carbon-coated zirconium dioxide fiducial very similar size to SC (5 mm × 1 mm) and used in a similar fashion [28]. In a small feasibility study of 11 patients, 3–5 of these carbon fiducials were placed at the margins of the lumpectomy cavity. They were reported to be well visualised on planning CT and cause less metal artifact than SC or gold markers. They are limited by the limited number of ‘points’ compared to sHA gel insertion. Surgical complication rate data was not reported.

TraceIT® (polyethylene glycol (PEG) radiopaque hydrogel, Augmenix Inc, Bedford, MA), Neauvia Organic Intense (hyaluronic acid hydrogel, MatexLab, Lugano, Switzerland), and BioXmark® (low-viscous solution composed of sucrose acetate isobutyrate (SAIB), electron dense SAIB analogue and ethanol, Nanovi, Copenhagen, DK), are all tissue fiducial markers that we assume can be used in a similar fashion to the sHA gel in our study. Surgical complication rate data was only reported for TraceIT in a small study of 24 patients in the interventional group, which was associated with 9.5 % rate of surgical site infection, which is similar to our study. Whilst Wiercinska et al. [29] also investigated a hyaluronic acid hydrogel, their methodology relied upon concurrent presence and use of SC placed at the deep margin of the cavity to aid in TB delineation using CT. In our future TB delineation study, we compare the use of sHA gel alone visualised by T2 weighted MRI versus use of clips alone visualised by CT.

VeraForm® (Videra Surgical inc, USA) is a radiopaque surgical filament that is threaded into the tumour cavity walls, adaptable to the specific surgical procedure performed, creating a 3-dimensional target for RO to delineate. In a small study of 20 patients, Mitchell et al. [30] reported no infections and no removal of the filament marker. This appears to be the most promising marker so far in terms of overcoming disadvantages of the other markers plus being visible on CT. Although it would not be visible if MRI were used in the delivery of RT.

## Conclusions

Use of sHA gel as a novel marker for the post WLE TB cavity in early breast cancer has been shown to be surgically feasible in this prospective study of thirty-five patients. We eagerly await further evaluation of its usefulness in TB delineation on MRI planning scans to establish true clinical applicability.

## CRediT authorship contribution statement

**Janice Yeh:** Conceptualization, Methodology, Investigation, Writing - original draft, Writing - review & editing, Visualization, Funding acquisition. **Grace Chew:** Methodology, Investigation. **Suat Li Ng:** Methodology, Investigation. **Wei Ming Ooi:** Methodology, Investigation. **Su-Wen Loh:** Methodology, Investigation, Funding acquisition. **Anthony Hyett:** Methodology, Investigation, Writing - review & editing. **Tristan Leech:** Methodology, Investigation. **Elaine Bevington:** Methodology, Investigation. **Jenny Huynh:** Methodology, Investigation. **Jenny Sim:** Supervision, Writing - review & editing. **Farshad Foroudi:** Supervision, Conceptualization, Methodology, Writing- review & editing, Resources. **Sweet Ping Ng:** Supervision, Methodology, Writing - review & editing. **Michael Chao:** Supervision, Conceptualization, Methodology, Investigation, Writing - review & editing, Funding acquisition, Project administration, Resources.

## Declaration of competing interest

The authors declare the following financial interests/personal relationships which may be considered as potential competing interests: ‘Michael Chao reports equipment, drugs, or supplies was provided by Palette Life Sciences. Michael Chao reports a relationship with Palette Life Sciences that includes: consulting or advisory.’.
